# Targeting Ferroptosis to Restore Salivary Gland Homeostasis in an Obesity Model

**DOI:** 10.3390/ijms27010514

**Published:** 2026-01-04

**Authors:** Gi Cheol Park, Hanaro Park, Soo-Young Bang, Ji Min Kim, Sung-Chan Shin, Yong-il Cheon, Ha-Nee Kwon, Jung Hwan Cho, Byung-Joo Lee

**Affiliations:** 1 Department of Otolaryngology—Head and Neck Surgery, Samsung Changwon Hospital, Sungkyunkwan University School of Medicine, Changwon 51353, Republic of Korea; uuhent@skku.edu (G.C.P.); naronaro@skku.edu (H.P.); 2 Department of Otorhinolaryngology—Head and Neck Surgery, College of Medicine, Pusan National University and Biomedical Research Institute, Pusan National University Hospital, Busan 49241, Republic of Korea; sooyoungbang@pusan.ac.kr (S.-Y.B.);; 3 Divisions of Endocrinology and Metabolism, Department of Internal Medicine, Samsung Changwon Hospital, Sungkyunkwan University School of Medicine, Changwon 51353, Republic of Korea; 4 Department of Otorhinolaryngology—Head and Neck Surgery, Good Gang-An Hospital, Busan 48265, Republic of Korea

**Keywords:** ferroptosis, obesity, salivary gland, mitochondrial dysfunction, ferrostatin-1, deferoxamine

## Abstract

Obesity is a systemic metabolic disorder that is known to impair various organ systems; however, its precise impact on salivary gland homeostasis remains unclear. Recent studies have implicated ferroptosis—an iron-dependent form of regulated cell death characterized by lipid peroxidation and oxidative stress—in glandular dysfunction. In this study, we used leptin-deficient (*ob*/*ob*) mice to elucidate the role of ferroptosis in obesity-associated salivary gland pathology. The protective effects of ferroptosis inhibition were evaluated by administering ferrostatin-1 (a lipid reactive oxygen species [ROS] scavenger) and deferoxamine (an iron chelator) for an 8-week period. Obese mice exhibited significantly increased body weight, food intake, and hyperglycemia. These systemic changes are accompanied by profound histological alterations in the salivary glands, including lipid droplet accumulation, acinar atrophy, and mitochondrial ultrastructural damage. These alterations correlate with the hallmarks of ferroptotic injury, including increased ROS levels (*p* < 0.001), elevated malondialdehyde levels (*p* < 0.01), suppressed glutathione peroxidase 4 activity (*p* < 0.01), and iron overload (*p* < 0.001). Salivary gland fibrosis, inflammation, and secretory dysfunction were evident, characterized by the upregulation of TGF-β (*p* < 0.01) and Collagen I (*p* < 0.05), reduced expression of aquaporin-5 and amylase, and dysregulated levels of autophagy-related markers (LC3B and p62). Treatment with either ferrostatin-1 or deferoxamine significantly mitigated these pathologies; however, the degree of efficacy varied depending on the specific parameters that were examined. Thus, our findings implicate ferroptosis as a critical contributor to salivary gland dysfunction in obesity and suggest that pharmacological inhibition of this pathway represents a viable therapeutic strategy for preserving glandular integrity under metabolic stress.

## 1. Introduction

Obesity is a rapidly escalating global health crisis that is characterized by generalized metabolic and inflammatory disturbances [1]. Despite the extensive characterization of systemic complications, the impact of obesity on salivary gland homeostasis remains poorly understood [2,3]. Newer evidence indicates that obesity is associated with reduced salivary secretion, altered glandular composition, and an increased susceptibility to oral dysfunction. Leptin-deficient *ob*/*ob* mice, which develop profound hyperphagia and severe obesity, serve as robust models for elucidating the pathological mechanisms underlying obesity-induced salivary gland dysfunction [4,5,6].

Among the diverse forms of regulated cell death, ferroptosis has recently attracted attention as a key driver of tissue injury in metabolic and inflammatory diseases [7]. Ferroptosis, which is characterized by iron-dependent accumulation of lipid peroxides that frequently occurs with the depletion or inactivation of glutathione peroxidase 4 (GPX4), causes overwhelming oxidative damage to polyunsaturated fatty acid-containing membranes. Morphologically distinct from apoptosis or necrosis, ferroptosis is characterized by unique mitochondrial ultrastructural changes, including cristae collapse, outer membrane rupture, and increased matrix density [8,9,10].

Salivary glands are highly metabolic organs that are particularly vulnerable to oxidative stress. Given their essential roles in oral homeostasis, antimicrobial defense, and digestive function, salivary gland dysfunction has clinically meaningful consequences that extend beyond local oral symptoms, particularly under chronic metabolic stress conditions [11]. Under various pathological conditions, ferroptosis plays a central role in salivary gland dysfunction. In a study that used ovariectomized rat models, treatment with ferrostatin-1 effectively preserved the glandular architecture and suppressed lipid peroxidation [12,13,14,15]. Subsequent studies extended these findings to radiation-induced salivary gland injury, wherein compounds such as amifostine and melatonin exert protective effects by inhibiting the ferroptotic processes [16]. The evaluation of ferroptosis in hepatic tissues revealed a critical role for ferritinophagy, particularly Nuclear Receptor Coactivator 4 (NCOA4)-mediated pathways, in promoting steatotic liver injury [17].

Despite these advances, the specific contribution of ferroptosis in obesity-driven salivary gland pathology remains largely unknown. Although both ferrostatin-1 (a lipid radical scavenger) and deferoxamine (an iron chelator) are established modulators of ferroptotic pathways, their comparative efficacy in mitigating obesity-associated salivary gland injury remains unelucidated.

This study investigated the involvement of ferroptosis in salivary gland dysfunction in obesity and evaluated the therapeutic potential of ferrostatin-1 and deferoxamine-induced regulatory effects in a mouse model. Utilizing comprehensive histological, biochemical, and ultrastructural analyses, we aimed to elucidate the mechanistic underpinnings of ferroptosis in obese salivary glands and assess the comparative efficacy of the abovementioned two pharmacological inhibitors in restoring glandular homeostasis.

## 2. Results

### 2.1. Obesity-Associated Changes in Weight, Nutrient Intake, and Glycemic Status

**Figure 1:**
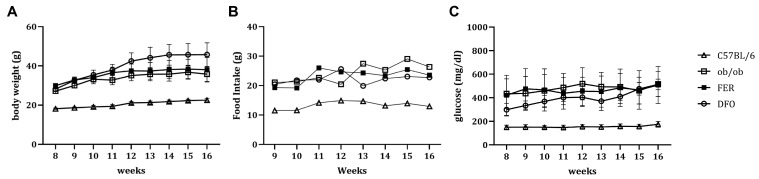
General physiological parameters in C57BL/6, *ob*/*ob*, FER, and DFO groups. (**A**) Body weight, (**B**) daily food intake, and (**C**) fasting blood glucose levels were measured in C57BL/6 control, untreated *ob*/*ob*, and *ob*/*ob* mice treated with either ferrostatin-1 (FER) or deferoxamine (DFO) throughout the experimental period. FER—ferrostatin-1-treated *ob*/*ob* mice; DFO—deferoxamine-treated *ob*/*ob* mice.

Body weight was consistently elevated in all obese groups, including untreated *ob*/*ob* mice, compared to that in C57BL/6 control mice (Figure 1A). Moreover, the daily food intake significantly increased across these groups and reflected the characteristic leptin deficiency-associated hyperphagic behavior (Figure 1B). In parallel, fasting glucose levels were markedly elevated in the untreated *ob*/*ob* group, and remained similarly high in the FER and DFO groups throughout the experimental period (Figure 1C). These systemic metabolic alterations established a physiologically unfavorable environment that potentially fosters subsequent histological and functional deterioration of the salivary glands.

### 2.2. Enhanced Lipogenesis in the Salivary Glands of Obese Mice

**Figure 2:**
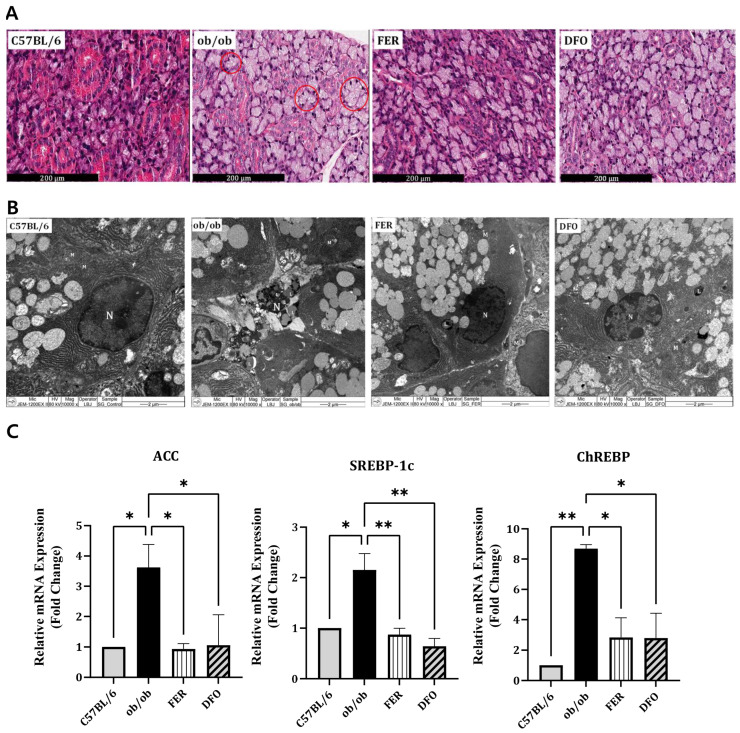
Lipid accumulation in the salivary glands of C57BL/6, *ob*/*ob*, FER, and DFO groups. (**A**) Representative H&E-stained images showing structural changes in the salivary glands. Lipid droplet-like cytoplasmic vacuoles are indicated by red circles in the *ob*/*ob* group. (**B**) Transmission electron microscopy images illustrating cytoplasmic lipid droplets (L), mitochondria (M), nuclei (N), and glandular components (G). (**C**) Relative mRNA expression levels of ACC, SREBP-1c, and ChREBP in the salivary glands that were analyzed by qPCR. Elevated expression in the *ob*/*ob* group was normalized to control levels following treatment with ferrostatin-1 or deferoxamine. * *p* < 0.05, ** *p* < 0.01. *n* = 4. *Columns* and *error bars* represent the mean ± standard deviation. FER—ferrostatin-1-treated *ob*/*ob* mice; DFO—deferoxamine-treated *ob*/*ob* mice.

Histological analysis revealed pronounced acinar atrophy, expanded interstitial spaces, and extensive cytoplasmic vacuolization that was consistent with lipid-droplet accumulation in the salivary glands of untreated *ob*/*ob* mice (Figure 2A). Transmission electron microscopy (TEM) further confirmed the presence of abundant cytoplasmic lipid droplets within acinar cells. Notably, this accumulation was markedly reduced in both DFO- and FER-treated groups (Figure 2B). At the transcriptional level, *ob*/*ob* mice exhibited significantly elevated expression of key lipogenesis-related genes, including *acetyl-CoA carboxylase (ACC)*, *sterol regulatory element-binding protein-1c (SREBP-1c)*, and *carbohydrate-responsive element-binding protein (ChREBP)*. Treatment with either ferrostatin-1 or deferoxamine substantially suppressed the expression of these lipogenic genes (Figure 2C). Collectively, these findings demonstrate that obesity promotes profound lipid accumulation in the salivary glands, as evident at the histological, ultrastructural, and molecular levels, which can be effectively mitigated by ferroptosis-targeted interventions. Additional representative histological and immunohistochemical images from independent sections are provided in Supplementary Figure S1.

### 2.3. Ferroptosis-Associated Oxidative Stress and Iron Dysregulation

**Figure 3:**
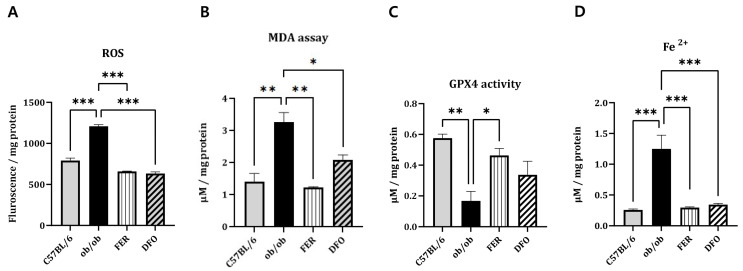
Oxidative stress and iron accumulation associated with ferroptosis in the salivary glands of C57BL/6, *ob*/*ob*, FER, and DFO groups. (**A**) Reactive oxygen species (ROS) levels, which were measured using a DCFH-DA fluorescence assay, were significantly elevated in the *ob*/*ob* group and decreased following treatment with either ferrostatin-1 or deferoxamine. (**B**) Malondialdehyde (MDA) levels increased in the *ob*/*ob* mice and markedly decreased by ferrostatin-1, rather than by deferoxamine, treatment. (**C**) GPX4 enzymatic activity was reduced in the *ob/ob* group and more effectively restored in the FER group compared to the DFO group. (**D**) Cytosolic ferrous iron (Fe^2+^) levels were elevated in the *ob*/*ob* mice and normalized in both treatment groups. * *p* < 0.05, ** *p* < 0.01, *** *p* < 0.001. *n* = 4. *Columns* and *error bars* represent mean ± standard deviation. FER—ferrostatin-1-treated *ob*/*ob* mice; DFO—deferoxamine-treated *ob*/*ob* mice; GPX4—glutathione peroxidase 4.

To investigate whether obesity-induced salivary gland injury involves ferroptosis, we measured the key indices of oxidative damage and iron accumulation. ROS levels were significantly elevated in the *ob*/*ob* group, as determined by DCFH-DA fluorescence assay, and were effectively reduced by either ferrostatin-1 or deferoxamine (*p* < 0.001) (Figure 3A). Moreover, the MDA content—a marker of lipid peroxidation—increased in the *ob*/*ob* group. Ferrostatin-1 treatment induced a greater reduction in MDA levels than the deferoxamine treatment (*p* < 0.01 and *p* < 0.05, respectively) (Figure 3B). Similarly, the primary ferroptosis defense mechanism, indicated by GPX4 enzymatic activity, was markedly impaired in *ob*/*ob* mice. This activity was more robustly restored by ferrostatin-1 than by deferoxamine (*p* < 0.05) (Figure 3C). In contrast, cytosolic Fe^2+^ levels, which were significantly elevated in the *ob*/*ob* group, were comparably reduced by either treatment (*p* < 0.001) (Figure 3D). These results confirmed that obesity promotes ferroptosis-associated oxidative stress and iron overload in salivary glands. Furthermore, they suggest that, compared with deferoxamine, ferrostatin-1 exerts superior efficacy in mitigating lipid peroxidation and restoring antioxidant capacity in this model.

### 2.4. Obesity-Induced Fibrosis and Inflammation in the Salivary Glands

**Figure 4:**
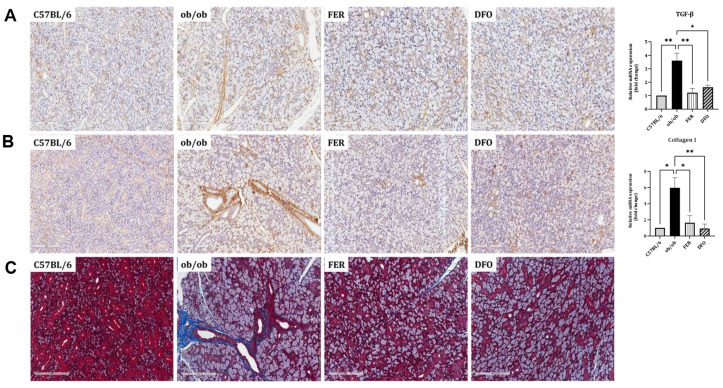
Histological and transcriptional assessment of profibrotic and stress-responsive markers in obese salivary glands. Immunohistochemical staining and mRNA expression analysis were performed to assess fibrotic and inflammatory changes in the salivary glands of C57BL/6, *ob*/*ob*, DFO, and FER groups. (**A**,**B**) TGF-β and Collagen I expression were increased in the *ob*/*ob* group. These proteins were visualized as blue-stained structures using a blue chromogen, and their reduction following treatment was confirmed by both immunostaining and qPCR. Ferrostatin-1 suppressed TGF-β expression more effectively, whereas deferoxamine more markedly reduced Collagen I. (**C**) Masson’s trichrome (MT) staining was performed to visualize collagen deposition (indicated by blue-stained fibers). The *ob*/*ob* group showed extensive interstitial fibrosis, which was comparably reduced by both treatments. * *p* < 0.05, ** *p* < 0.01. *n* = 4. *Columns* and *error bars* represent the mean ± standard deviation. FER—ferrostatin-1-treated *ob*/*ob* mice; DFO—deferoxamine-treated *ob*/*ob* mice.

To evaluate whether obesity-induced ferroptosis is accompanied by fibrotic remodeling, we analyzed the histological and transcriptional markers in the salivary glands. In the *ob*/*ob* group, immunohistochemical staining revealed the increased expression of the profibrotic mediators TGF-β and Collagen I. These were visualized as blue-stained structures using a blue chromogen, indicating enhanced fibrotic activity. This increase was attenuated in both treatment groups. The qPCR analysis corroborated these histological findings. TGF-β mRNA was significantly upregulated in the *ob*/*ob* group and was more robustly suppressed by ferrostatin-1 than by deferoxamine (*p* < 0.01 and *p* < 0.05, respectively). In contrast, Collagen I mRNA expression showed a stronger suppressive response to deferoxamine (*p* < 0.01; Figure 4A,B). Masson’s trichrome (MT) staining was performed to visualize collagen deposition. The *ob*/*ob* group exhibited extensive interstitial fibrosis, characterized by diffuse blue-stained collagen fibers, which were markedly reduced following either ferrostatin-1 or deferoxamine treatment (Figure 4C). These findings indicate that ferroptosis is closely linked to fibrotic remodeling in salivary glands under obese conditions, with ferrostatin-1 and deferoxamine exhibiting differential regulatory effects on specific fibrosis-related pathways. Additional representative histological and immunohistochemical images from independent sections are provided in Supplementary Figure S1.

### 2.5. Functional Deterioration of the Salivary Glands and Its Reversal by Ferroptosis Inhibitors

**Figure 5:**
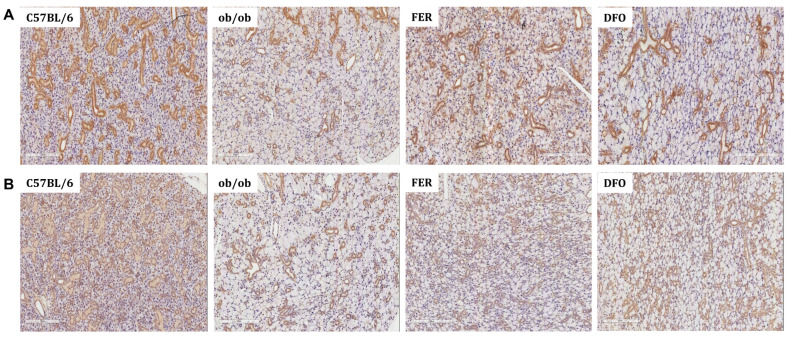
Expression of salivary gland functional proteins α-amylase and AQP5 in C57BL/6, *ob*/*ob*, DFO, and FER groups. (**A**) Immunohistochemical staining of α-amylase showed prominent cytoplasmic expression in the acinar cells of C57BL/6 mice, reduced staining in the *ob*/*ob* group, and restored levels following treatment, with stronger recovery observed in the FER group. (**B**) AQP5 staining was localized to the apical membrane of acinar cells and was markedly reduced in the *ob*/*ob* mice; however, this was restored in both treatment groups, with ferrostatin-1 inducing a more prominent effect. FER, ferrostatin-1-treated *ob*/*ob* mice; DFO, deferoxamine-treated *ob*/*ob* mice.

To assess the secretory function of salivary glands, we performed immunohistochemical staining for functional markers. In the *ob*/*ob* group, the expression of α-amylase—a key digestive enzyme—was markedly reduced compared to that in C57BL/6 controls. This reduction was partially reversed in both treatment groups; however, a more pronounced recovery was observed in the FER group (Figure 5A). Similarly, the expression of aquaporin-5 (AQP5)—a water-channel protein that is essential for saliva secretion—decreased in the *ob*/*ob* group. Compared to deferoxamine treatment, ferrostatin-1 treatment more effectively restored AQP5 expression (Figure 5B). These findings suggest that obesity-induced ferroptosis significantly impairs salivary gland secretory function and that pharmacological inhibition of this pathway, particularly with ferrostatin-1, can effectively facilitate functional recovery. Additional representative histological and immunohistochemical images from independent sections are provided in Supplementary Figure S1.

### 2.6. Mitochondrial Dysfunction and Selective Autophagy Imbalance in the Obese Salivary Glands

**Figure 6:**
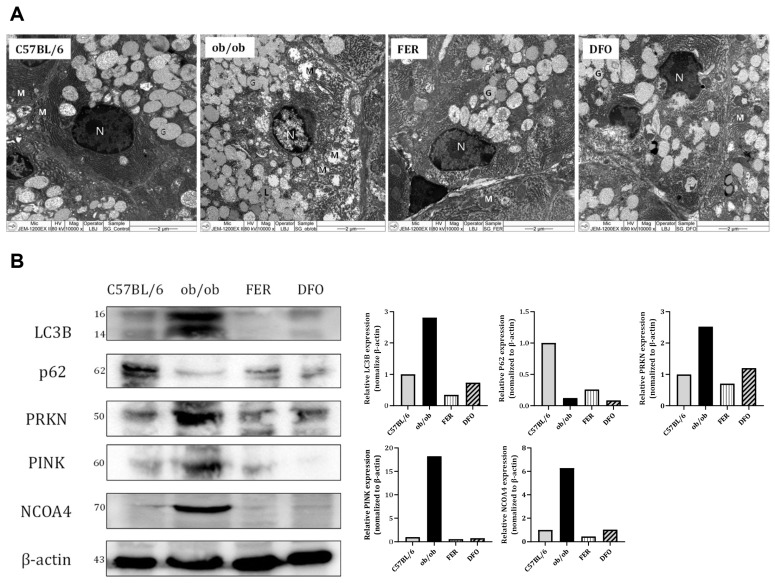
Mitochondrial ultrastructure and selective autophagy markers in salivary glands. (**A**) Transmission electron microscopy (TEM) images of the salivary glands showing mitochondrial morphology in each group. The *ob*/*ob* group exhibited extensive mitochondrial swelling, cristae loss, and outer membrane disruption; however, these alterations were alleviated in the FER and DFO groups. Cytoplasmic lipid droplets (L), mitochondria (M), nuclei (N), and glandular components (G). (**B**) Western blot analysis of autophagy- and mitophagy-related proteins. LC3B expression was markedly increased in the *ob*/*ob* group and reduced following treatment. In contrast, p62 levels were lowest in the *ob*/*ob* group and partially restored by both ferrostatin-1 and deferoxamine. PINK1 and PRKN were elevated in the *ob*/*ob* group and decreased following treatment, with PINK1 expression better maintained in the FER group. NCOA4 was highly expressed in the *ob*/*ob* group but was suppressed in all other groups. FER—ferrostatin-1-treated *ob*/*ob* mice; DFO—deferoxamine-treated *ob*/*ob* mice.

Considering the observed phenotypic changes, we examined the mitochondrial ultrastructure and regulation of selective autophagy pathways. In the *ob*/*ob* group, TEM revealed severe mitochondrial ultrastructural damage, which was characterized by swelling, loss of cristae, and outer membrane rupture. These morphological changes were partially mitigated by ferrostatin-1 or deferoxamine treatment (Figure 6A). Western blotting revealed distinct alterations in key autophagy- and mitophagy-regulatory proteins. The expression of LC3B-II (the lipidated form, indicated at 14 kDa) was most prominently induced in the *ob*/*ob* group. Concurrently, the levels of p62 (SQSTM1)—an autophagy substrate—were markedly decreased in *ob*/*ob* mice compared with controls. This pattern (high LC3B-II and low p62 levels) suggested enhanced autophagic degradation (increased flux) in the obese state. Both treatments reduced LC3B-II levels and partially restored p62 levels, which suggested the normalization of autophagic flux. The mitophagy regulators PINK1 and PRKN (parkin) were upregulated in the *ob*/*ob* group. Either treatment decreased the expression of these proteins. Notably, ferrostatin-1 maintained PINK1 expression at levels that were similar to those in the control whereas deferoxamine resulted in a near-complete loss of the PINK1 signal. NCOA4—a cargo receptor for ferritinophagy—is selectively elevated in *ob*/*ob* mice, which suggests increased ferritin turnover. NCOA4 levels were suppressed in both the control and treatment groups (Figure 6B). These findings indicate that obesity-induced salivary gland pathology involves significant mitochondrial damage and the dysregulation of selective autophagy pathways, including mitophagy and ferritinophagy. The modulation of autophagic flux and mitophagy markers by ferroptosis inhibitors, particularly the preservation of PINK1 by ferrostatin-1, likely contributed to the observed tissue-protective effects.

## 3. Discussion

This study provided compelling evidence that obesity-induced salivary gland dysfunction is associated with ferroptosis. In the *ob*/*ob* mouse model, this is evidenced by pronounced lipid peroxidation, iron dysregulation, mitochondrial damage, and impaired secretory function. Through integrated histological, biochemical, ultrastructural, and molecular assessments, we established that ferroptosis plays a pivotal role in salivary gland pathology under metabolic stress. Critically, the pharmacological inhibition of ferroptosis using either ferrostatin-1 or deferoxamine significantly attenuated these pathological changes, which validated ferroptosis as a viable therapeutic target for obesity-associated salivary gland disorders.

Ectopic lipid accumulation has emerged as a prominent feature of salivary gland injury in obese mice that suggests lipotoxicity [5,18,19,20]. Acinar atrophy, cytoplasmic vacuolization, and TEM-confirmed lipid droplets were accompanied by the transcriptional upregulation of key lipogenic regulators, including *SREBP-1c*, *ACC*, and *ChREBP*. These results are consistent with those of hepatic steatosis models, which suggested that salivary glands undergo a similar maladaptive lipogenic shift in response to chronic metabolic overload [17]. Both ferroptosis inhibitors effectively suppressed lipogenic gene expression, and this indicates a close relationship between lipid dysregulation and ferroptotic stress. This suggests the presence of a potential feedback loop wherein oxidative stress exacerbates lipid synthesis, which, in turn, fuels further ferroptosis by increasing the availability of substrates for peroxidation.

The cardinal hallmarks of ferroptosis, oxidative stress, and iron accumulation were evident in the salivary glands of the obese mice [21,22,23]. Elevated ROS and MDA levels, reduced GPX4 activity, and increased cytosolic Fe^2+^ collectively confirmed the activation of the ferroptotic cascade. Compared with deferoxamine, ferrostatin-1 demonstrated a superior capacity to suppress lipid peroxidation (MDA) and restore GPX4 activity; however, both treatments comparably reduced cytosolic iron levels. These findings suggest that obesity fosters a lipid ROS-driven ferroptotic milieu in salivary glands. Consequently, the direct neutralization of lipid radicals (by ferrostatin-1) appears to confer broader cytoprotection rather than iron chelation monotherapy (by deferoxamine).

Furthermore, obesity induced substantial fibrotic remodeling, as indicated by the increased expression of TGF-β and Collagen I, and confirmed by MT staining. Interestingly, ferrostatin-1 more effectively reduced *TGF-β* expression whereas deferoxamine more prominently suppressed *Collagen I* transcripts. This divergence suggests that ferroptosis contributes to fibrotic signaling through complex pathways that involve both iron-and lipid peroxide-dependent mechanisms. These further highlights that different ferroptosis inhibitors can differentially modulate the distinct downstream nodes of tissue remodeling [24,25,26,27].

Salivary acinar cells are the primary functional units responsible for the production and secretion of saliva, which comprises water, electrolytes, and digestive enzymes such as amylase. Since these cells are essential for oral homeostasis and the initiation of digestion, their structural and functional impairment directly compromises salivary gland function. Functionally, the diminished expression of α-amylase and AQP5 in obese salivary glands reflects a significant impairment in both the production of digestive enzymes and fluid-secretion capacity. The expression of both proteins was restored by ferrostatin-1 or deferoxamine treatment; however, ferrostatin-1 consistently demonstrated superior restorative efficacy. These results align with those of previous studies, which demonstrated that ferroptosis impairs secretory epithelial function in hormone-deficient and radiation-induced salivary gland injury. This study is the first to significantly extend this paradigm to the context of metabolic diseases [12,13,15,16].

Although the present study focuses on salivary glands, ferroptosis has been implicated in dysfunction of other secretory and metabolic organs under obese conditions, including the liver [17]. Salivary glands, however, are characterized by continuous secretory activity, high mitochondrial density, and pronounced redox sensitivity, which may confer increased susceptibility to ferroptotic injury. Therefore, while ferroptosis represents a shared pathological mechanism across organs, its functional consequences are likely to be tissue-specific.

The mitochondrial ultrastructure exhibited classical ferroptosis-associated damage, including swelling, cristae disruption, and outer membrane rupture, which confirmed mitochondrial vulnerability [8,28,29,30]. Autophagy and mitophagy markers revealed complex patterns of dysregulation. The combination of increased LC3B-II and decreased p62 levels suggests an enhanced autophagic flux (increased degradation) in the obese state. Concurrently, the upregulation of PINK1 and PRKN indicates heightened mitophagy activation, likely in response to mitochondrial damage. Furthermore, the selective elevation of NCOA4 expression implies the induction of ferritinophagy, which contributes to increased intracellular free iron levels. Treatment modulated these markers, albeit with distinct profiles for the two inhibitors. Ferrostatin-1 preserved PINK1 expression at near-control levels, whereas deferoxamine almost completely abolished this effect. This striking difference suggests divergent effects on the mitochondrial quality control mechanisms and turnover dynamics. Taken together, these findings underscore the intricate intersection between ferroptosis, selective autophagy, and mitochondrial homeostasis in the functional deterioration of salivary glands under obese conditions [31,32].

A key strength of this study was the direct comparative analysis of ferrostatin-1 and deferoxamine—two canonical ferroptosis inhibitors with distinct mechanisms of action [33,34,35]. Ferrostatin-1 functions as a lipophilic radical-trapping antioxidant that directly neutralizes lipid peroxyl radicals and thereby inhibits LPO propagation. In contrast, deferoxamine sequesters redox-active Fe^2+^ through chelation and indirectly mitigates ferroptotic stress by limiting Fenton chemistry-mediated ROS generation. These mechanistic differences translate into different biological outcomes. Ferrostatin-1 exhibited superior efficacy in reducing MDA, restoring GPX4 activity, preserving mitophagy-related protein expression (e.g., PINK1), and improving functional markers (AQP5 and amylase). In contrast, deferoxamine exerted a stronger suppressive effect on *Collagen I* mRNA expression. These findings indicate that a specific point of intervention within the ferroptotic cascade critically dictates the downstream tissue responses, which highlights the necessity for mechanism-specific therapeutic strategies that are tailored to the underlying pathology.

This study had some limitations. First, functional salivary flow measurements, which would have provided direct evidence for secretory restoration, were not performed. Second, the long-term and potential off-target effects of chronic ferroptosis inhibition warrant further investigation. Third, the upstream regulators of ferroptosis susceptibility, such as NRF2, ACSL4, and SLC7A11, have not been examined yet. Fourth, while leptin-deficient mice provide a robust and reproducible platform for investigating metabolic-driven injury, this genetic model may not fully capture the multifactorial nature of human obesity seen in diet-induced obesity (DIO) models. Further studies using DIO models are warranted to validate these ferroptotic mechanisms in a more diverse physiological context. Investigating these factors may provide deeper mechanistic insights into the metabolic–redox interface that governs salivary gland ferroptosis.

## 4. Materials and Methods

### 4.1. Animal Models and Experimental Design

Leptin-deficient (*ob*/*ob*) mice, characterized by excessive food intake and pronounced obesity, are widely used as robust models of metabolic disorders. In this study, we used *ob*/*ob* mice to investigate the contribution of ferroptosis to obesity-induced alterations in the salivary glands. Six-week-old female C57BL/6-Jms Slc mice (*n* = 8) and age-matched C57BL/6J *ob*/*ob* mice (n = 24) were obtained from Japan SLC (Shizuoka, Japan). Following a 2-week acclimation period, the animals were randomly assigned to four groups (n = 8 per group) as follows: wild-type C57BL/6 (control), untreated *ob*/*ob*, ferrostatin-1-treated *ob*/*ob* (FER), and deferoxamine-treated *ob*/*ob* (DFO). All animals were fed standard chow ad libitum throughout the study period. Beginning at 8 weeks of age, mice in the FER and DFO groups received intraperitoneal (IP) injections of ferrostatin-1 (5 µM/kg) or deferoxamine (100 mg/kg), respectively, three times weekly for 8 weeks. The control and untreated *ob*/*ob* groups received vehicle (sterile saline) injections according to the same schedule. To monitor pathological progression, baseline and endpoint assessments were conducted at 8 and 16 weeks of age. At the study endpoint, the animals were anesthetized via isoflurane inhalation and blood samples were collected from the inferior vena cava. Subsequently, the mice were euthanized and salivary gland tissues were harvested for subsequent analysis. All experimental procedures were approved by the Institutional Animal Care and Use Committee of the Pusan National University Hospital (IACUC approval no. PNUH-2023-223).

### 4.2. Measurement of Lipid Peroxidation

The level of malondialdehyde (MDA), a biomarker of lipid peroxidation, was quantified using a commercial detection kit (Abcam, Cambridge, UK). Tissue homogenates and standards were incubated with thiobarbituric acid (TBA) at 95 °C for 1 h. Following rapid cooling on ice, the absorbance of the MDA-TBA adduct was spectrophotometrically measured at 532 nm. The MDA concentrations were determined by interpolating a standard curve.

### 4.3. Cytosolic Iron Quantification

Intracellular ferrous iron (Fe^2+^) levels were assessed using a colorimetric assay kit (Abcam, ab83366). Approximately 30 mg of salivary gland tissue was homogenized in Iron Assay Buffer and centrifuged at 16,000× *g* for 10 min at 4 °C. The supernatant was reacted with an iron probe under acidic conditions to produce a colored complex, which was detected spectrophotometrically at 593 nm. Data were normalized to the total protein content that was determined using the bicinchoninic acid (BCA) assay.

### 4.4. Detection of Reactive Oxygen Species (ROS)

ROS generation was measured using the fluorescent probe 2′,7′-dichlorodihydrofluorescein diacetate (DCFH-DA; Sigma-Aldrich, St. Louis, MO, USA). Homogenized tissue samples were incubated with 10 µM DCFH-DA at 37 °C for 30 min in the dark. Oxidation of the probe yielded fluorescent dichlorofluorescein (DCF), which was measured fluorometrically (e.g., using a fluorescence microplate reader) at excitation/emission wavelengths of 485/535 nm. The fluorescence intensity was normalized to the protein concentration.

### 4.5. GPX4 Activity Assay

Glutathione peroxidase 4 (GPX4) activity was evaluated using a commercial assay kit (Abcam). Enzymatic activity was quantified by measuring the rate of NADPH oxidation. Protein concentrations were determined using the BCA method, and activity was calculated according to the manufacturer’s protocol.

### 4.6. Histological and Immunohistochemical Analysis

Salivary glands were excised immediately after euthanasia, fixed overnight in 4% formalin, and paraffin-embedded. Because mouse salivary glands are very small, multiple specimens from animals within the same experimental group were embedded together and stained on the same slide to ensure uniform staining conditions and to allow direct comparison across samples. Serial paraffin sections were prepared so that histologic and immunohistochemical analyses could be performed at anatomically comparable levels among animals.

Sections were stained with hematoxylin and eosin (H&E; Sigma-Aldrich, St. Louis, MO, USA) for general morphology or Masson’s trichrome (MT; Sigma-Aldrich, St. Louis, MO, USA) for fibrosis assessment. For immunohistochemistry (IHC), deparaffinized sections underwent antigen retrieval by heating in 10 mM sodium citrate buffer (pH 6.0; sodium citrate buffer (pH 6.0)) for 15 min using a microwave oven, followed by cooling at room temperature (23 °C). Sections were then incubated with horseradish peroxidase-conjugated secondary antibodies (ENZO Biochem Inc., New York, NY, USA) for 1 h at room temperature (23 °C), followed by visualization using the chromogen 3,3′-diaminobenzidine (DAB; Vector Laboratories, Burlingame, CA, USA) and counterstaining with hematoxylin. Negative controls were processed in parallel by substituting PBS containing 1% BSA for the primary antibody.

Representative images were acquired from the central region of each gland using a Leica light microscope at 200× magnification. For each animal, 3–4 serial sections were evaluated, and 5–7 microscopic fields per section were analyzed in a fully blinded manner to ensure methodological rigor.

### 4.7. Transmission Electron Microscopy (TEM)

For ultrastructural analysis, salivary gland tissues were pre-fixed in 2.5% glutaraldehyde (Sigma-Aldrich, St. Louis, MO, USA) in phosphate buffer and post-fixed in 1% osmium tetroxide (Electron Microscopy Sciences, Hatfield, PA, USA). After dehydration through a graded ethanol series, samples were embedded in Epon 812 resin. Ultrathin sections (50–60 nm) were stained with uranyl acetate and lead citrate and examined using a JEOL JEM-1200EXII (JEOL Ltd., Tokyo, Japan) transmission electron microscope.

To ensure adequate quantitative assessment of mitochondrial morphology, three animals per group were examined. For each animal, 2–3 ultrathin sections were prepared, and 8–10 TEM fields per section were analyzed. Mitochondrial integrity was assessed in a blinded manner, focusing on cristae structure, membrane continuity, and the degree of swelling.

### 4.8. Quantitative Real-Time PCR

Total RNA was extracted using the TRIzol reagent (Thermo Fisher Scientific, Waltham, MA, USA). Complementary DNA (cDNA) synthesis was synthesized using a reverse transcription kit (Applied Biosystems, Foster City, CA, USA). Quantitative real-time PCR (qPCR) was conducted on an ABI PRISM 7900 HT system using SYBR Green Chemistry. The relative gene expression levels were calculated using the 2^−ΔΔCt^ method, with *GAPDH* normalized as the endogenous reference gene. The primer sequences are listed in Table 1.

### 4.9. Western Blotting

Tissue lysates were prepared in a radioimmunoprecipitation assay (RIPA) buffer that was supplemented with protease and phosphatase inhibitor cocktails (Roche, Basel, Switzerland). Lysates were cleared by centrifugation at 12,000× *g* for 15 min at 4 °C. Proteins were separated using sodium dodecyl sulfate–polyacrylamide gel electrophoresis (SDS-PAGE) and transferred onto nitrocellulose membranes. These membranes were blocked (e.g., with 5% non-fat milk) and incubated overnight at 4 °C with the following primary antibodies: anti-LC3B, anti-p62, anti-PRKN, anti-PINK1, and anti-NCOA4 (all from Abcam, Cambridge, UK; 1:1000 dilution), and anti-β-actin (Sigma-Aldrich, St. Louis, MO, USA). HRP-conjugated secondary antibodies (1:5000) were applied for 2 h at room temperature. Signals were detected using an enhanced chemiluminescence (ECL) reagent and visualized using an imaging system.

### 4.10. Statistical Analysis

All data are presented as mean ± standard deviation (SD). Intergroup comparisons were performed using one-way analysis of variance (ANOVA) followed by Scheffé’s post hoc test using SPSS (v19.0; IBM Corp., Armonk, NY, USA). A *p*-value < 0.05 was considered as the cutoff for statistical significance.

## 5. Conclusions

This study established ferroptosis as a critical driver of salivary gland dysfunction in a leptin-deficient mouse model of obesity. Pharmacological inhibition using ferrostatin-1 or deferoxamine effectively mitigates oxidative stress, suppresses lipogenic gene expression, attenuates fibrosis, and restores salivary gland function. Although both agents conferred protection, they exhibited distinct molecular profiles and differential efficacies, particularly in the modulation of lipid peroxidation, iron homeostasis, and autophagic pathways. Collectively, these findings highlight the potential of ferroptosis as a promising therapeutic strategy for managing obesity-associated salivary gland disorders.

## Figures and Tables

**Table 1:** Primer sequences used for qPCR analysis.

GENE	Sequence (5′3′)	
	Forward	Reverse
GAPDH	AGCCCAAGATGCCCTTCAGT	CCGTGTTCCTACCCCCAATG
SREBP-1c	ACGGAGCCATGGATTGCACA	AAGGGTGCAGGTGTCACCTT
ChREBP	CTGGGGACCTAAACAGGAGC	GAAGCCACCCTATAGCTCCC
ACC	ATGGGCGGAATGGTCTCTTTC	TGGGGACCTTGTCTTCATCAT
TGF-β1	GTGTGGAGCAACATGTGGAACTCTA	TTGGTTCAGCCACTGCCGTA
Collagen I	CCTCAGGGTATTGCTGGACAAC	CAGAAGGACCTTGTTTGCCAGG

## Data Availability

The original contributions presented in this study are included in the article/Supplementary Materials. Further inquiries can be directed to the corresponding author.

## Supplementary Materials

The following supporting information can be downloaded at: https://www.mdpi.com/article/10.3390/ijms27010514/s1.

## Abbreviations

ACC—acetyl-CoA carboxylase; AQP5—aquaporin 5; ChREBP—carbohydrate-response element-binding protein; DAB—3,3′-diaminobenzidine; DFO—deferoxamine; Fer-1—ferrostatin-1; GPX4—glutathione peroxidase 4; H&E—hematoxylin and eosin; IHC—immunohistochemistry; LC3B—microtubule-associated proteins 1A/1B light chain 3B; LPO—lipid peroxidation; MDA—malondialdehyde; MT—Masson’s trichrome; NCOA4—nuclear receptor coactivator 4; p62—sequestosome-1; PINK1—PTEN-induced kinase 1; PRKN—Parkin; ROS—reactive oxygen species; SREBP-1c—sterol regulatory element-binding protein-1c; TEM—transmission electron microscopy; TGF-β—transforming growth factor-beta; 4-HNE—4-hydroxynonenal.
