# Supplementary material for: Targeting Ferroptosis to Restore Salivary Gland Homeostasis in an Obesity Model
Source: Int J Mol Sci. 2026 Jan 4;27(1):514. doi: 10.3390/ijms27010514 (PMC12786536; doi:10.3390/ijms27010514)

Additional supplementary figure for Figure 2A

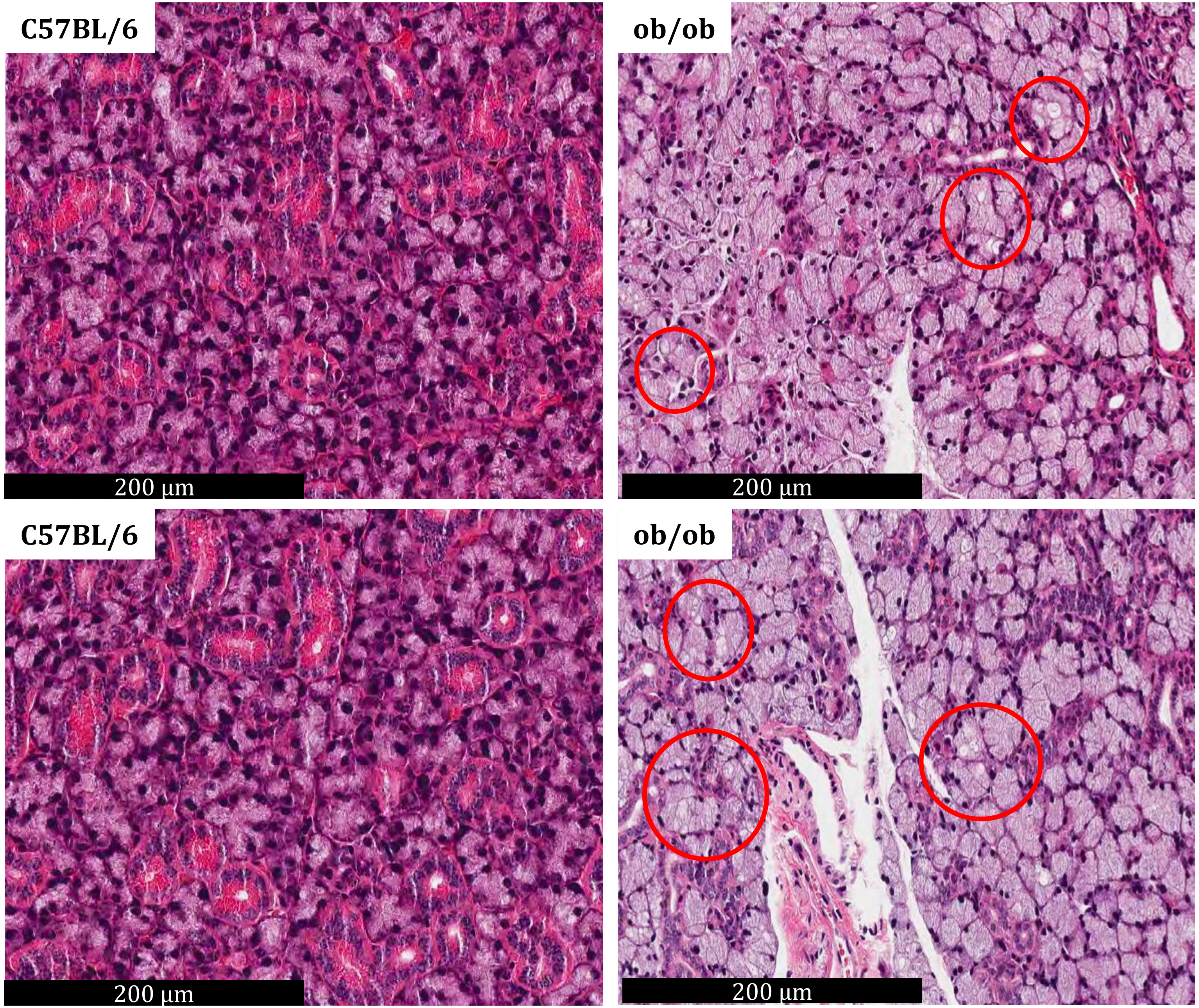
C57BL/6
200 µm
ob/ob
200 µm
C57BL/6
200 µm
ob/ob
200 µm

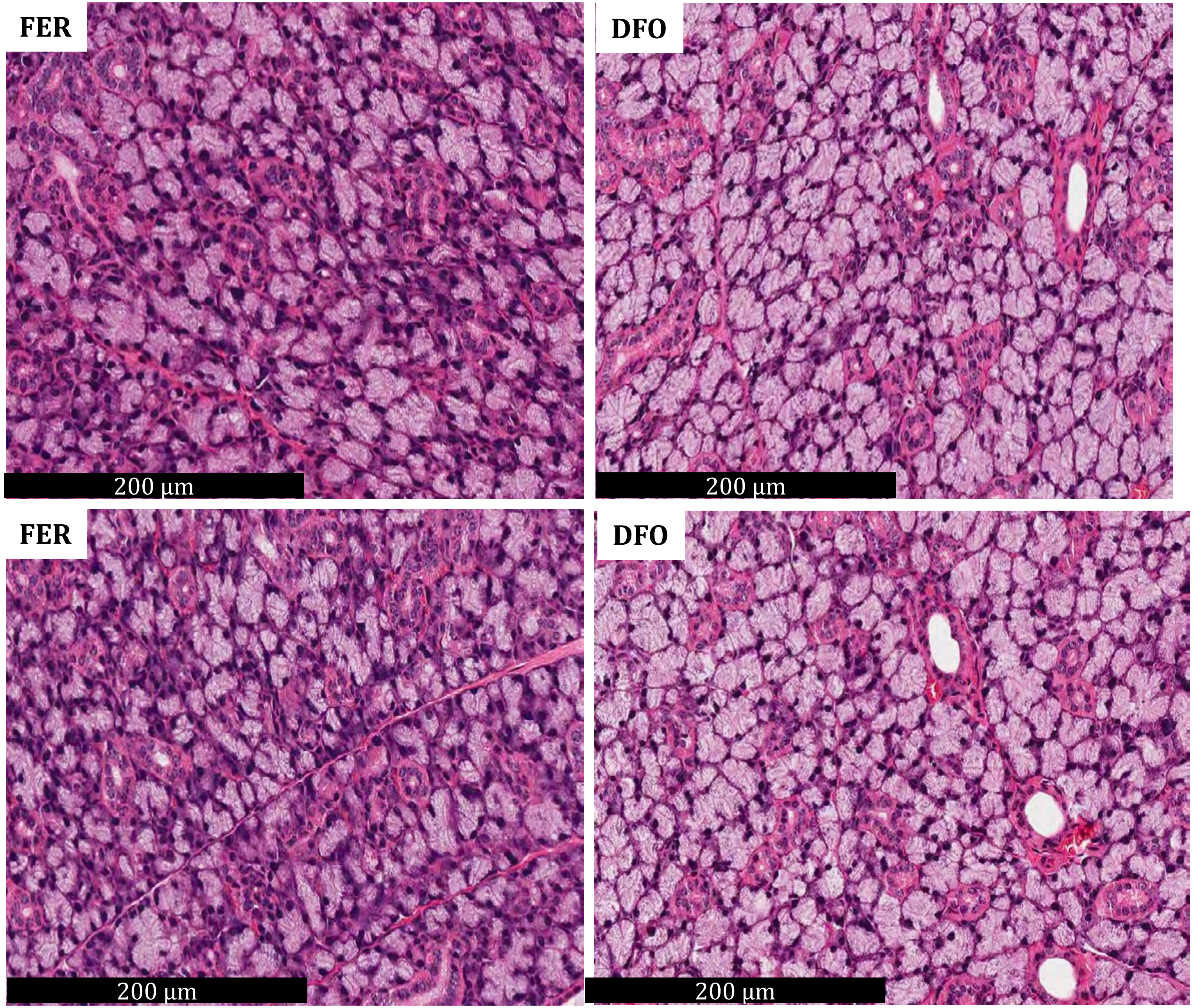
FER
200 µm
DFO
200 µm
FER
200 µm
DFO
200 µm

Additional supplementary figure for Figure 4A

TGF-β

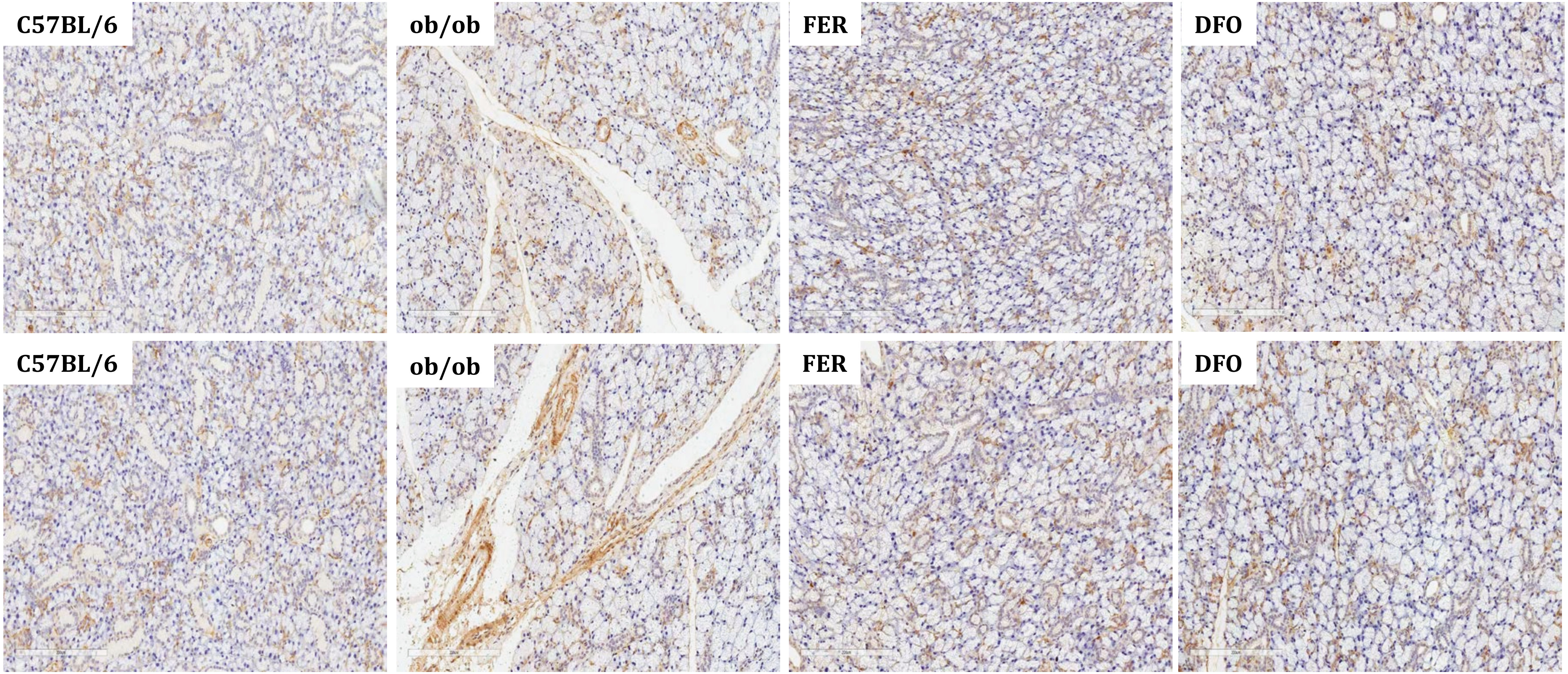

Additional supplementary figure for Figure 4B

Collagen I

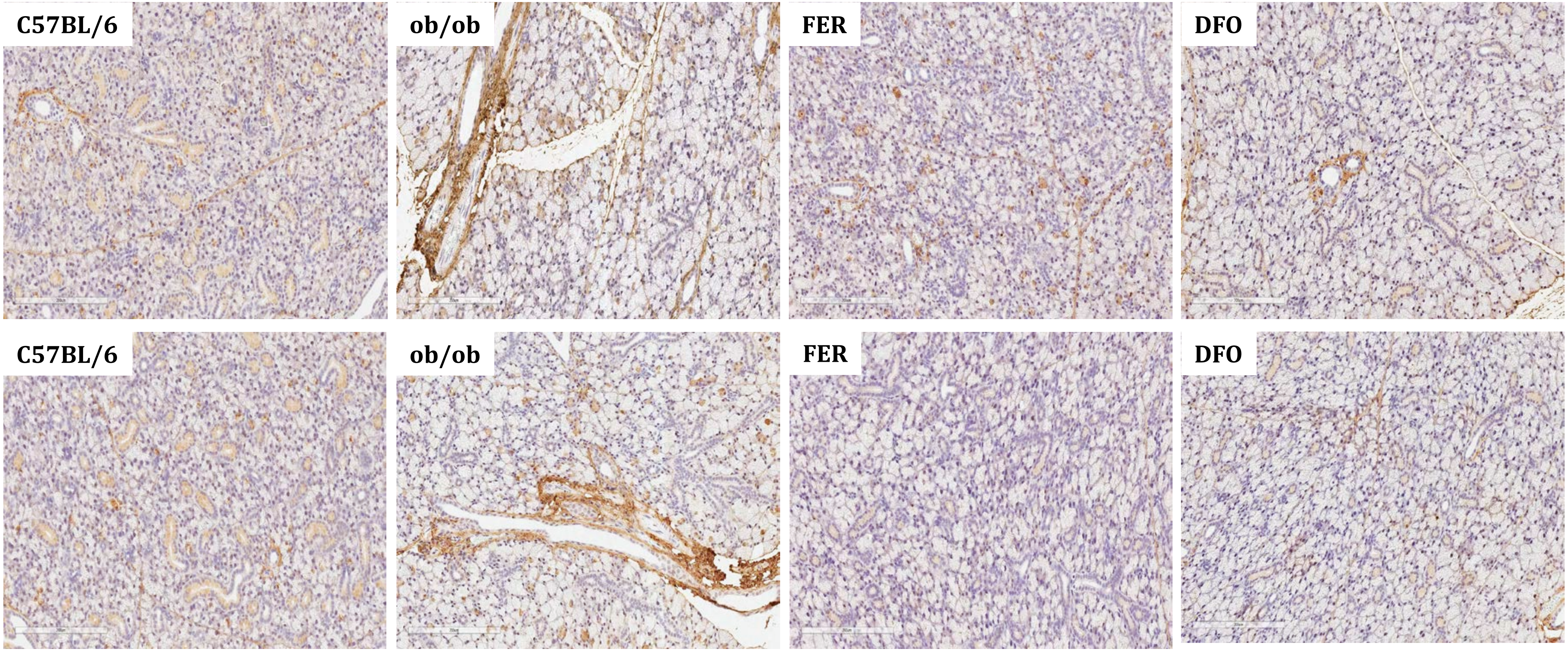

Additional supplementary figure for Figure 4C

MT

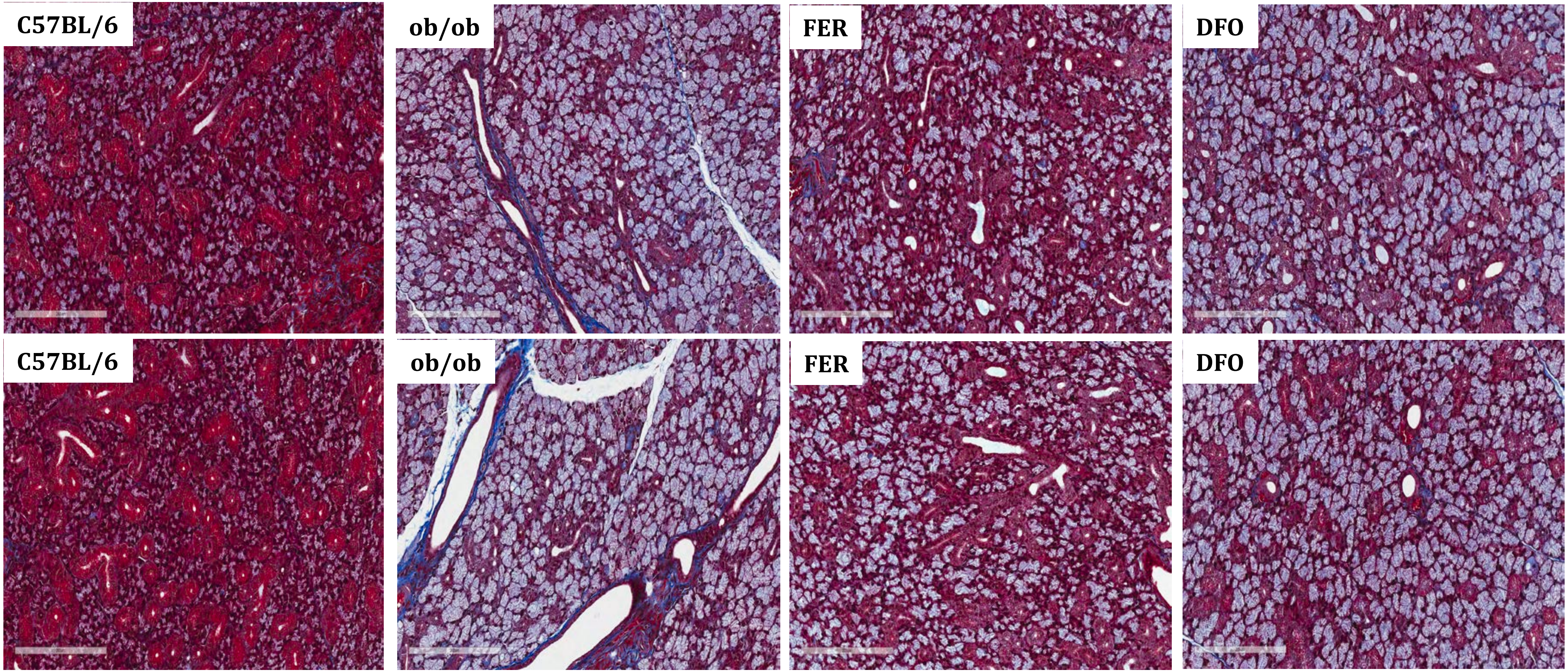

Additional supplementary figure for Figure 5A

α – amylase

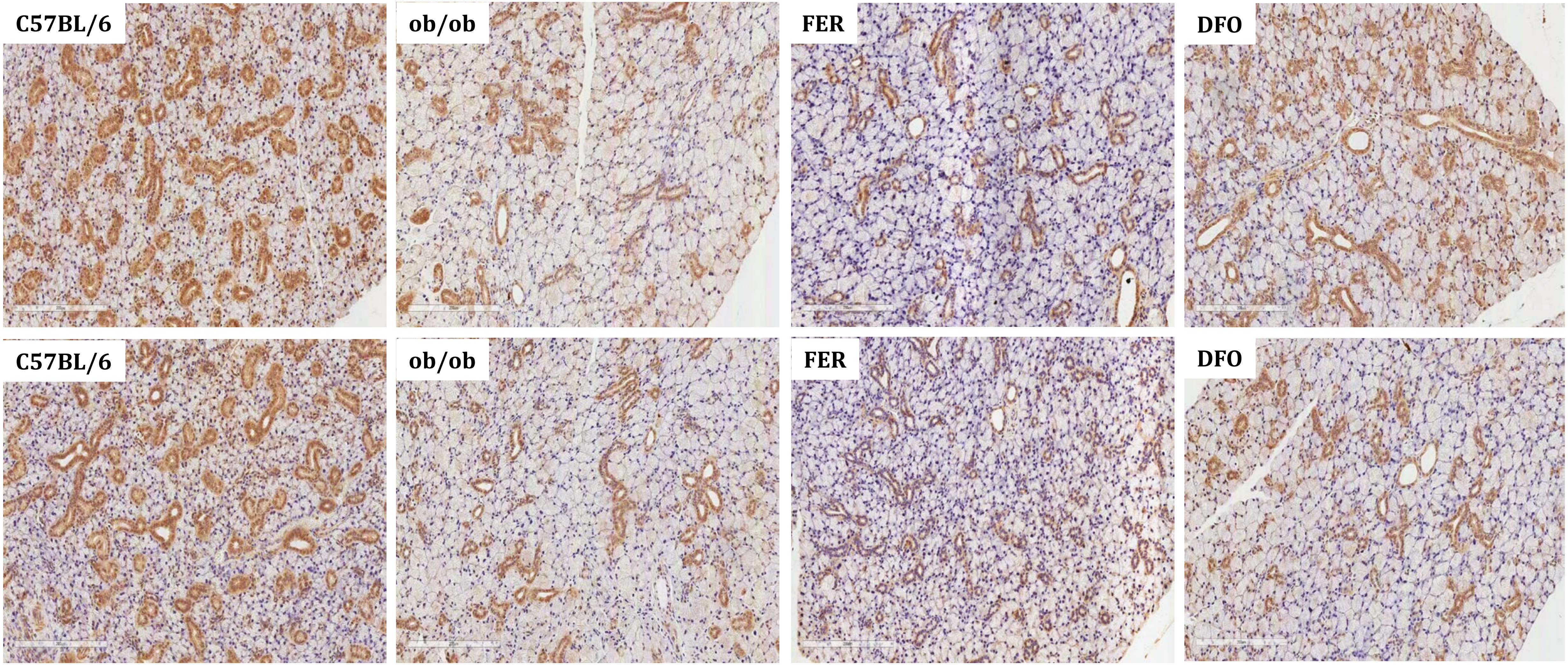

Additional supplementary figure for Figure 5B

Aqp-5

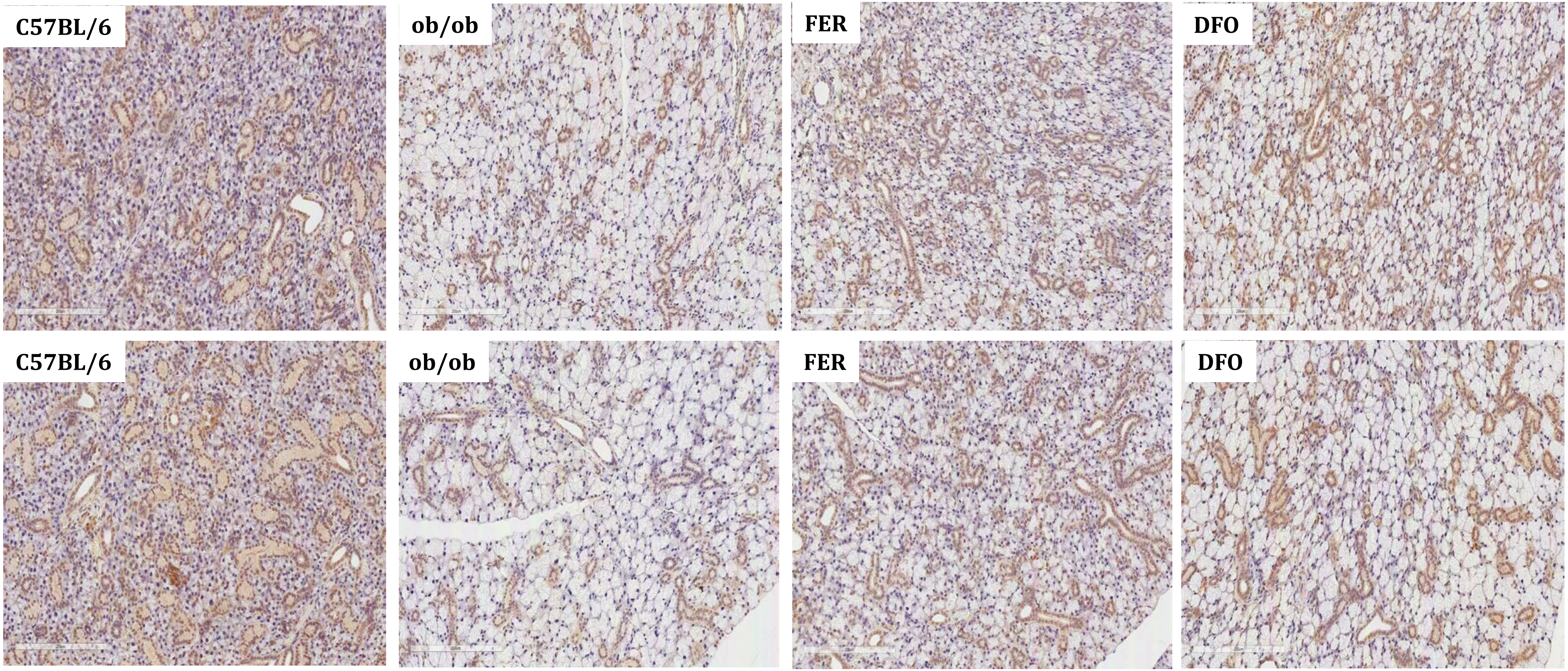

Supplement: Supplementary file 1 [file ijms-27-00514-s001.zip › ijms-4062511-supplementary.pdf]
